# Editorial: Nuclear receptors in hemodynamics and blood pressure control

**DOI:** 10.3389/fphys.2023.1290411

**Published:** 2023-09-27

**Authors:** John D. Imig, Jing Wu, Eman Y. Gohar

**Affiliations:** ^1^ Department of Pharmaceutical Sciences, University of Arkansas for Medical Sciences, Little Rock, AR, United States; ^2^ Department of Medicine, Institute for Human Health and the Environment, University of Rochester Medical Center, Rochester, NY, United States; ^3^ Department of Pharmacology and Physiology, Institute for Human Health and the Environment, University of Rochester Medical Center, Rochester, NY, United States; ^4^ Vanderbilt University Medical Center, Nashville, TN, United States

**Keywords:** nuclear receptors, blood pressure, hemodynamics, peroxisome proliferator-activated receptors, farnesoid X-activated receptor

This Research Topic in *Frontiers in Physiology* focused on emerging research on nuclear receptors and cardiovascular function in health and disease. Unlike cell membrane G-protein-coupled receptors that elicit biological responses through second messenger signaling, nuclear receptors are a large family of transcription factors that primarily bind to genomic DNA and regulate the expression of target genes, although non-genomic actions of nuclear receptors have also been identified (Bishop-Bailey, 2015). Nuclear receptors typically consist of a ligand-binding domain, a DNA-binding domain, and a hinge domain that together interact with hormone response elements at DNA transcription regulation sites and transactivation domain, leading to nuclear receptor dimerization. Ligand binding at nuclear receptors triggers a cascade of molecular events that invariably lead to molecular regulation to activate or suppress transcription of target genes.

In response to endogenous or exogenous ligands such as lipids, vitamins, and hormones, nuclear receptors regulate a wide range of biological processes, including growth, development, metabolism, homeostasis, and reproduction (Bishop-Bailey, 2015). The natural nuclear receptor ligands are small hydrophobic molecules, which makes designing their structural analogs as pharmacological nuclear receptor modulators easy. These selective and non-selective nuclear receptor agonists and antagonists have been developed for research purposes and are used in the treatment of human diseases (Kumar and Narkar, 2022). Nuclear receptors play critical roles in the control of organ blood flow and blood pressure. Steroid hormone receptors and peroxisome proliferator-activated receptors (PPARs) are well-established regulators of sympathetic outflow, cardiac output, vascular function, kidney water and electrolyte transport, inflammation, and other physiological responses (Bishop-Bailey, 2015; Fang et al., 2021). This Research Topic presents two review articles and two original articles that highlight new insights into the physiological functions of nuclear receptors in controlling hemodynamics and blood pressure.

Two original research articles provide novel findings on the regulation of angiotensin type 1 (AT1) receptors by the estrogen metabolite, 2-methoxyestradiol (2ME2), and quantification of turbulent flow and aortic disease development. Zhang et al. demonstrated the therapeutic anti-hypertensive potential of 2ME2 in angiotensin-hypertensive and spontaneously hypertensive rats (SHRs). These studies demonstrate that 2ME2 downregulates AT1 receptor expression in the kidney cortex and liver without impacting angiotensin II binding affinity (Zhang et al.). The blood pressure-lowering effect of 2ME2 is independent of sex in Wistar rats infused with angiotensin II. In addition, prolonged 2ME2 treatment decreases heart rate and body weight in male SHRs. Collectively, this study demonstrated that 2ME2 plays a critical role in regulating the renin–angiotensin system and resting heart rate through the downregulation of angiotensinogen and AT1 receptor (Zhang et al.). Sundin et al. determined that turbulent kinetic energy of blood flow in the healthy human thoracic aorta increases with dobutamine stress and is strongly related to cardiac output. Quantification by magnetic resonance imaging (MRI) was performed to determine 4D flow-based hemodynamic parameters and turbulent kinetic energy and evaluate dobutamine stress on thoracic aortas. Findings of this study demonstrate that turbulent kinetic energy with cardiac stress could serve as a risk assessment for aortic disease development (Sundin et al.). Future studies that focus on the therapeutic potential of 2ME2 to treat hypertension and MRI-based 4D turbulent kinetic measurement to assess cardiovascular disease progression are required.

Two review articles in this Research Topic focused on PPAR and farnesoid X receptor (FXR) therapeutics for hypertension and PPARγ regulation in salt-sensitive hypertension and insulin resistance. Imig focused on PPAR and FXR dual modulating drugs for the treatment of metabolic diseases, organ fibrosis, and hypertension. Evidence from clinical studies and animal hypertension models demonstrated that PPAR and FXR agonism can lower blood pressure and decrease end-organ damage in metabolic diseases and hypertension. This review article details the emerging dual modulating drugs that combine PPAR and FXR agonism with soluble epoxide hydrolase (sEH) inhibition or Takeda G-protein receptor 5 (TGR5) agonism (Imig). In preclinical studies, these novel drugs demonstrate reduced side effects in addition to anti-hypertensive, anti-fibrotic, and anti-inflammatory actions. Ertuglu et al. focused on PPARγ regulation in salt-sensitive hypertension and insulin resistance. This review highlights the ability of PPARγ agonists to increase insulin sensitivity and ameliorate salt sensitivity. These findings on the role for PPARγ in pathogenesis of insulin sensitivity and salt sensitivity were coupled with newly found effects on the immune system and vascular function (Ertuglu et al.). These review articles provide a framework for future studies to explore nuclear receptor-based drugs in treating metabolic and cardiovascular diseases.

The Research Topic *Nuclear Receptors in Hemodynamics and Blood Pressure Control* presents two original articles and two review articles that highlight emerging findings in the field that could lead to enhanced cardiovascular risk detection, therapeutics for hypertension including salt-sensitive hypertension, and development of dual modulating drugs for metabolic and cardiovascular diseases. Continued understanding of the contribution of nuclear receptors to controlling organ blood flow and blood pressure is needed for the development of effective therapies to combat hypertension and other cardiovascular diseases.

## References

[B1] Bishop-Bailey D. (2015). Nuclear receptors in vascular biology. Curr. Atheroscler. Rep. 17 (5), 507. 10.1007/s11883-015-0507-8 25772409

[B2] Fang S. Livergood M. C. Nakagawa P. Wu J. Sigmund C. D. (2021). Role of the peroxisome proliferator activated receptors in hypertension. Circ. Res. 128 (7), 1021–1039. 10.1161/CIRCRESAHA.120.318062 33793338 PMC8020861

[B3] Kumar A. Narkar V. A. (2022). Nuclear receptors as potential therapeutic targets in peripheral arterial disease and related myopathy. FEBS J. 10.1111/febs.16593 PMC9908775 35942640

